# Craniodental anatomy in Permian–Jurassic Cynodontia and Mammaliaformes (Synapsida, Therapsida) as a gateway to defining mammalian soft tissue and behavioural traits

**DOI:** 10.1098/rstb.2022.0084

**Published:** 2023-07-03

**Authors:** Luke A. Norton, Fernando Abdala, Julien Benoit

**Affiliations:** ^1^ Evolutionary Studies Institute, University of the Witwatersrand, Private Bag 3, Wits 2050, Johannesburg, South Africa; ^2^ Unidad Ejecutora Lillo, CONICET-Fundación Miguel Lillo, Miguel Lillo 251, Tucumán 4000, Argentina

**Keywords:** mammal evolution, hair, lactation, secondary palate, mastication, parietal foramen

## Abstract

Mammals are diagnosed by more than 30 osteological characters (e.g. squamosal-dentary jaw joint, three inner ear ossicles, etc.) that are readily preserved in the fossil record. However, it is the suite of physiological, soft tissue and behavioural characters (e.g. endothermy, hair, lactation, isocortex and parental care), the evolutionary origins of which have eluded scholars for decades, that most prominently distinguishes living mammals from other amniotes. Here, we review recent works that illustrate how evolutionary changes concentrated in the cranial and dental morphology of mammalian ancestors, the Permian–Jurassic Cynodontia and Mammaliaformes, can potentially be used to document the origin of some of the most crucial defining features of mammals. We discuss how these soft tissue and behavioural traits are highly integrated, and how their evolution is intermingled with that of craniodental traits, thus enabling the tracing of their previously out-of-reach phylogenetic history. Most of these osteological and dental proxies, such as the maxillary canal, bony labyrinth and dental replacement only recently became more easily accessible—thanks, in large part, to the widespread use of X-ray microtomography scanning in palaeontology—because they are linked to internal cranial characters.

This article is part of the theme issue ‘The mammalian skull: development, structure and function’.

## 1. Introduction

**Figure 1.**
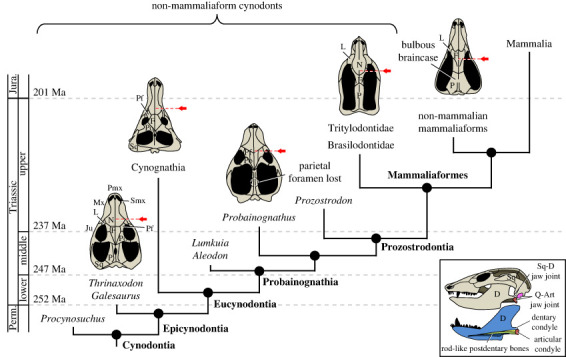
Simplified cladogram of the Cynodontia and cranial morphology in dorsal view (modified from Ruta *et al.* [11]). Note the simplification of the circumorbital region and the posterior migration of the internal choanae compared to the orbit (posterior extension of the secondary palate, marked by a red arrow). Inset: skull and mandible of a cynodont in lateral view (top) and lower jaw in medial view (bottom) illustrating the dual jaw joint condition. Art, articular; D, dentary; F, frontal; J, jugal; Jura., Jurassic; L, lacrimal; Mx, maxilla; N, nasal; P, parietal; Perm., Permian; Pf, prefrontal; Pmx, premaxilla; Po: postorbital; Q: quadrate; Smx: septomaxilla; Sq: squamosal. Drawings not to scale. (Online version in colour.)

Craniodental traits, such as the presence of a large lower temporal fenestra and enlarged caniniform teeth that separate the anterior and posterior dentition, are pivotal for tracing the origin of modern mammals among the Permian–Jurassic synapsids. Mammalia can be identified in the fossil record by the occurrence of over 37 osteological characters, including a squamosal-dentary jaw articulation, ossification of the maxillary turbinates, reduction of the postdentary bones to form the inner ear ossicles, reduction or loss of the coronoids and absence of quadratojugals and tabulars [1]. By contrast, the clade Mammalia has traditionally been characterized by a suite of predominantly physiological, soft tissue and behavioural traits, including endothermy, the presence of fur, mammary glands, extensive parental care, an isocortex and endothermy [1–10]. Direct evidence for these characters is rarely preserved in the fossil record, making the task of tracing their evolutionary origins in mammalian forebears, i.e. the mostly Permian–Jurassic non-mammaliaform cynodonts (NMC) and their descendants the non-mammalian mammaliaforms (NMM), quite difficult (figure 1). Recent works have nevertheless shown that these soft tissue and behavioural traits are directly related to nervous structures, sense organs and other osteological and dental structures that leave readily observable evidence on the fossilized skulls of these taxa [6–8,10]. This literature review paints an overview of the main cranial, mandibular and dental transformations from the NMC and NMM conditions to those of Mammalia, offering a unique gateway to the evolutionary history of the defining mammalian soft tissue and behavioural traits listed above. The osteological traits are discussed below in anterior–posterior order on the skull.

## 2. Tooth replacement and diphyodonty

Most placental mammals are diphyodont, i.e. have a maximum of two tooth generations (deciduous ‘milk’ teeth that are replaced by permanent ‘adult’ teeth), whereas the plesiomorphic state for Synapsida was an intense and uninterrupted dental replacement throughout life [12]. Mammals also developed a unique type of dental occlusion (accompanied by fleshy cheeks and chewing as indicated by the presence of a masseteric fossa on the dentary) [13]. Numerous studies investigating tooth replacement patterns in NMC and NMM have been undertaken, using X-ray microtomography (CT) scanning [14–16] and histological sectioning [17,18], and have had converging results. The NMC display a wide variety of conditions of dental succession [14–16], but there is no definite evidence of diphyodonty in NMC [14–16]. Although this style of tooth replacement has recently been proposed for the mammalian-proximate taxon *Brasilodon* [18], the evidence presented is not compelling, and previous studies have indicated that dental replacement was not diphyodont in that taxon [19,20]. The earliest evidence for the reduction in number of replacement generations to a finite number (oligophyodonty) has been identified in the canines of Permian–Triassic epicynodonts [15,16]; however, it is only in the early Jurassic NMM *Morganucodon* that true diphyodonty evolved [21]. A reduction in the number of replacement generations probably coincided with four evolutionary adaptations. First, the appearance of prismatic enamel, as the increased resource input of producing large enamel-covered teeth from a young age meant that shedding teeth frequently through ontogeny would have been wasteful [22,23]. Second, the appearance of nutritive lactation, as developing functional, adult-like teeth at birth was no longer a necessary condition for feeding [24,25]. Third, compared to the plesiomorphic alternate dental replacement pattern of NMC, diphyodonty improves precise occlusion, which possibly fuelled an elevated metabolic rate [24]. Finally, the attachment of teeth via a periodontal ligament (gomphosis)—which is the basal theriodont condition—rather than fusion of the tooth to the bone (ankylosis), allows the dentition to shift slightly through ontogeny, further facilitating the maintenance of precise dental occlusion [26,27]. The advent of precisely occluding dentition in the cynodont lineage is probably also linked to the transition to a ‘dual jaw joint’ (discussed below), which together probably helped improve chewing efficiency [28–31].

## 3. Palate and nasal cavity

In mammals, the secondary palate is a sheet of bone that separates the nasal and oral cavities [24]. It is formed by medially projecting palatal processes of the premaxillae, maxillae and palatine bones. A secondary palate was absent in early synapsids and the development of a bony secondary palate is one of the best-documented transitions in the evolution of the synapsid lineage [24,32,33].

Basal synapsids had an open palate, but Permian and early Triassic NMC (e.g. *Procynosuchus* and *Galesaurus*, respectively) began evolving medially projecting expansions of the premaxillae, maxillae and palatines that were probably connected medially by soft tissue (e.g. a cartilaginous or membranous sheet) [34–36]. An almost fully closed palate developed for the first time in the early Triassic epicynodont NMC (e.g. *Thrinaxodon*), but remained anteroposteriorly short and with a gap separating the palatal processes of the maxillae and palatines throughout ontogeny [37]. The mid-to-early late Triassic probainognathian NMC (e.g. *Lumkuia*, *Aleodon* and *Probainognathus*) were the first to evolve an elongated secondary palate that pushed the choanae far back in the mouth, beyond the level of the anterior margin of the orbit [38,39], resembling the mammalian condition (figure 1).

The palate of NMC also has enlarged transverse processes of the pterygoid that slide against the medial part of the mandibles during jaw adduction. Along with the paracanine fossa that receives the lower canine when the jaws are closed, this represents a major morphological constraint that funnels the mandible during occlusion. In comparison, mammals and most NMM have reduced pterygoid wings that do not reach the mandible and no paracanine fossa [40–42]. The only exceptions are the basal NMM *Morganucodon* and *Megazostrodon*, in which a paracanine fossa is present and the reduced transverse (hamular) process still presents a facet for the mandible [40–42].

The development of the secondary palate may initially have been for a structural reinforcement of the snout, with a marked increase in torsional strength and stiffness that later would have extreme importance with the development of occlusion and strong bites [43]. The bony palate also separates the food-processing part of the snout from the respiratory tract, which enables chewing for long periods of time without impeding breathing [24,32,33]. By contrast, diapsids that lack a secondary palate need to pause their breathing while eating [9]. That the secondary palate evolved primarily for chewing is supported by the parallel development of a similarly constructed secondary palate in baurioid therocephalians that coincidentally also evolved chewing, as evidenced by the enlarged masseteric fossa on the dentary and the presence of dental occlusion [44–46]. This ability to breathe and eat simultaneously probably allowed for an increased metabolic rate, ultimately enabling endothermy to evolve in the most derived NMC [24,47]. Furthermore, a fully enclosed buccal cavity allows a vacuum to be created, which is necessary to create a buccal pump for young to suckle, facilitating the nursing of young in early developmental stages [32,33]. This evolved pairwise with a more complex hyoid morphology in NMM compared to the simple rod-like morphology of NMC [48].

As the nasal cavity became physically and functionally separated from the mouth, elements of the nasal capsule began ossifying, particularly the turbinates. In mammals, the ossified olfactory and respiratory turbinates support the olfactory epithelium and help retain water and warmth by acting as a counter current exchanger [32,33,49–51]. Unquestionable evidence of ossified turbinates has never been found in NMC and NMM, though bone fragments have been reported in the nasal cavity of some tritylodontids, brasilodontids and NMM [42,52], but see [33]. The presence of ridges on the internal walls of the nasal cavity suggests that at least cartilaginous turbinates were present in NMC and NMM [49,50]. The origin of ossified respiratory turbinates is correlated to the evolution of endothermy [47], whereas that of the olfactory ones was linked to the onset of mammal-like olfactory capabilities [53,54]. Both types of ossified turbinates could have originated in NMC [52] or in NMM [53,55], but in the absence of reliable evidence, phylogenetic bracketing can only support that ossified turbinates evolved in the last common ancestor of Mammalia.

## 4. Circumorbital region

The region of the skull around the orbit has undergone simplification in mammals compared to their NMC ancestors which had more bones in the skull [56]. In mammals, the prefrontal, postfrontal and postorbital bones are absent. The last two of these bones were lost altogether with the postorbital bar in probainognathian NMC belonging to the Prozostrodontia (figure 1). Though it has lost the postorbital bar, *Prozostrodon* itself (the basal-most prozostrodontian) retains vestigial prefrontal and postorbital bones [57]. Many hypotheses may account for the loss of the postorbital bar as, for example, it leaves room for the expansion of enlarged jaw adductor musculature, removes possible weak points at the sutures between circumorbital bones (possible adaptation for increased chewing activity) or may be a by-product of cranial miniaturization or orbit orientation [58,59].

Mammals are also noted for the presence of a simple infraorbital foramen (IOF), whereas most NMC possess multiple supralabial foramina on the snout [60,61]. These foramina have been colloquially referred to as ‘whisker pits’ [62], because they have long been hypothesized to be the rooting points for vibrissae [63,64]. This assumption has, however, been consistently and compellingly disproven many times. Similar foramina are absent in mammals, but are present in a wide variety of sauropsids, including dinosaurs and extant lizards [7,60,65–67]. The ‘whisker pits’ of early synapsids are only partly homologous to the IOF of mammals. In the probainognathian NMC, the number of ‘whisker pits’ becomes gradually reduced until only three foramina (or three clusters of foramina) remain in the clade unifying *Probainognathus* and the Prozostrodontia [7,66]. Kermack *et al*. [40] numbered these IOF1–IOF3 in anterior–posterior order. Using CT-scanning, Benoit *et al*. [66] showed that only IOF2 connects to the infraorbital canal and is thus homologous to the mammalian IOF. Prozostrodontian NMC were the first to acquire a mammalian IOF [66,67], but note that some cynognathian NMC convergently evolved a similar structure [68].

The presence of an IOF is correlated to whisking behaviour, as the foramen gives passage to the sensory fibres of the infraorbital branch of the trigeminal nerve, which innervate the whiskers in mammals [69–71]. Whiskers are hair, and as such, the origin of the IOF in the Prozostrodontia, some 241 Ma, may be linked to the evolutionary origin of endothermy [7].

## 5. Parietal foramen

Synapsids originally possessed a ‘third eye’ or pineal eye—a photoreceptive organ that detects changes in ambient light, aiding the monitoring of biological rhythms and thermoregulatory behaviour—accommodated in an opening between the paired parietal bones on the skull roof, called the parietal (or pineal) foramen [7,8]. This parietal foramen is normally absent in modern mammals [72], and the only relict of the organ that occupied it is the pineal gland, which has lost its photoreceptive function in mammals [73–76]. The loss of the parietal foramen happened in NMC and was a gradual process [8,77]. The presence and morphology of the parietal foramen became inconsistent in the late Permian and early Triassic NMC (variably present or absent within and between species, slit-like in adults, or lost during ontogeny) and is completely and irreversibly lost in the Probainognathia [7,8].

It has been proposed that the loss of the parietal foramen was linked to a mutation of the homeogene *Msx2*, which coincidentally also partly controls the maintenance of hair and development of the mammary glands, as demonstrated by experiments undertaken on mice [7,78–81] (see discussion). The loss of the parietal foramen in NMC also indicates that the pineal eye was no longer required [77]. The photoreceptive function of the pineal eye might have been compensated by the appearance of the melanopsin-containing retinal ganglion cells, and/or its role in monitoring behavioural thermoregulation might have become redundant owing to the evolutionary origin of endothermy [8,77]. One last hypothesis is that the pineal eye became non-functional in NMC owing to increased nocturnality [82].

## 6. Braincase

Mammals have a well-ossified braincase that almost completely encapsulates the brain [83]. By contrast, early epicynodont NMC, such as *Thrinaxodon* and *Galesaurus*, have some of the most poorly ossified braincases of all synapsids. They lack an ossified cribriform (ethmoid) plate to separate the nasal and brain cavities, the orbitosphenoids are reduced to two thin plates only (suspended in cartilage), the epipterygoids (which would eventually become the mammalian alisphenoids) are not as anteroposteriorly expanded as in later mammals—which result in the interorbital space being mostly filled by cartilage, offering little protection to the forebrain [53,83–86]. Therefore, the braincase of basal NMC is widely opened laterally and anteroventrally.

The ossified portions of the orbitosphenoid and alisphenoid expanded gradually in parallel in the Cynognathia and Probainognathia. The mammalian condition of a fully laterally enclosed braincase is attained in the late Triassic and early Jurassic Brasilodontidae and Tritylodontidae [83,87–89], before the origin of mammaliaforms. This went pairwise with a simplification of the braincase. The supratemporal and tabular are lost in prozostrodontian NMC. Developmental studies showed that the mammalian interparietal is formed by the fusion of four elements that correspond to the interparietals medially and the tabulars laterally [90]. The prootic and opisthotic fuse to form the periotic in Brasilodontidae, Tritylodontidae and Mammaliaformes [24,91]. There is no undisputable evidence of an ossified cribriform plate in any NMC or NMM, and it is likely that it remained cartilaginous until the origin of Mammalia [32,92,93].

This gradual increase in the ossification of the braincase and isolation of the brain from the outside world coincided with the continued increase in encephalization in probainognathian NMC and Mammaliaformes [53,85,94]. Noticeably, the olfactory bulbs increased in size as the ossified portion of the orbitosphenoid, the bone that supports them, expanded in probainognathian NMC [94]. The cerebral hemispheres (primitively tubular and undifferentiated in NMC) also began expanding caudally and laterally, and the cerebellar vermis (primitively concealed in NMC) became large enough to be exposed dorsally in *Probainognathus* and more derived NMC and NMM [94]. The braincase acquires a bulbous aspect in NMC, which is usually interpreted as the origin of the mammalian isocortex (or neocortex) [53]. The six-layered isocortex is a distinguishing feature of Mammalia as it is not present in any other vertebrate group [95] and is crucial for cognitive abilities and sensory perception [2,3]. Therefore, its origin is often hypothesized to be correlated with that of sensory hair [53,96], and in this respect, it is noticeable that the lateral expansion of the cerebral hemispheres occurs in almost the same taxa as those that evolved an IOF, i.e. the prozostrodontian NMC (see above). However, it is important to note that no NMC or NMM display a well-defined rhinal fissure that normally marks the ventral expansion of the isocortex on the sidewalls of the braincase in mammals [83,92,93,97]. In the absence of a clear rhinal fissure, inferring the presence of an isocortex is extremely speculative, and it is possible that an isocortex is limited to Mammalia.

## 7. Postdentary bones

**Figure 2.**
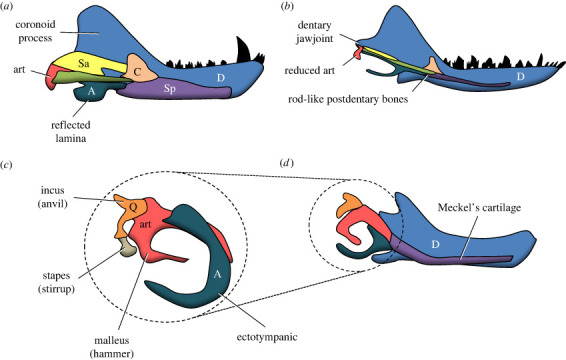
Evolution of the lower jaw, and the evolutionary and embryological transformation of the postdentary bones into the auditory ossicles. (*a*) Basal non-mammaliaform cynodont; (*b*) non-mammalian mammaliaform; (*c*) adult marsupial and (*d*) juvenile marsupial lower jaws in medial view. A, angular; Art, articular; C, coronoid; D, dentary; Pa, prearticular; Q, quadrate; Sa, surangular; Sp, splenial. Drawings not to scale. (Online version in colour.)

In NMC, the lower jaw includes seven different bones (figure 2*a*). The lower jaw articulation with the skull is ensured by two bones responsible for this function, the articular on the mandible and the quadrate on the cranium [98,99]. By contrast, the lower jaw in mammals is comprised entirely of the paired dentary bones, and the articulation with the skull is ensured by a dentary condyle (see below). Embryological and palaeontological evidence have shown that the stapes of NMC is homologous to the mammalian stirrup, the quadrate to the anvil (incus), the articular to the hammer (malleus) and the angular to the ectotympanic [24,100–107]. In mammals the articular and quadrate are still present, but are miniaturized, and have migrated to form part of the middle ear ossicular chain within the temporal cavity (figure 2*c,d*). Therefore, in mammals, the dentary articulates directly with the squamosal, with the quadrate and articular no longer playing a role in the jaw articulation, but in sound transmission instead [103,106]. Remarkably, many NMM and eucynodont NMC possess a ‘dual jaw-joint’ condition (figure 1), in which both the plesiomorphic quadrate-articular and derived squamosal-dentary complexes (or a transitional squamosal-surangular complex) are functional in the jaw articulation [44,105,106].

The changeover from the quadrate-articular articulation in NMC to the squamosal-dentary joint of mammals is among the most iconic and best-documented examples of macroevolutionary transitions in vertebrate palaeontology and embryology [24,100–107]. The transition is characterized by the gradual increase in size of the dentary bone and the reduction of the postdentary bones (i.e. articular, angular, coronoid, surangular and prearticular) across synapsids. The reduction of these postdentary bones into rod-like structures accommodated in a mandibular sulcus, or postdentary trough located medially to the large dentary, is a synapomorphy of Middle Triassic Eucynodontia [105]. A dual jaw joint is present since the Eucynodontia, but a distinct facet on the squamosal for the mandibular condyle appears only in the Prozostrodontia (with the noticeable exception of the Tritylodontidae) and *Probainognathus* [42]. Then, the quadrate-articular complex keeps on shrinking until it becomes less functionally important for the articulation of the lower jaw than the squamosal-dentary complex in the Mammaliaformes, in the late Triassic [108]. The transition towards the fully mammalian condition was completed when the postdentary bones (including the quadrate-articular complex) became completely detached from the lower jaw in adult individuals [109]. The presence or absence of a Meckelian groove on the lower jaw of Mesozoic NMM and mammals has shown that the transition from attached postdentary bones to detached auditory ossicles happened independently in monotremes, marsupials and placentals, as well as in five other lineages of mammaliaforms across the Mesozoic [105,110,111].

The precise factors that led to these complicated rearrangements and changes in lower jaw bones are not fully understood [112]. It has been proposed that the transition in function of the postdentary bones was driven as a means to improved efficiency in mastication [111–113]. The squamosal-dentary jaw articulation possibly allowed for a more rigid and stable jaw articulation to develop, facilitating the adaptation to exploit and chew food items more efficiently. Meanwhile, the enlargement of the dentary offered a more robust and broader surface of attachment for the jaw adductor muscles (e.g. the pterygoid muscles in mammaliaforms, [114]), thus increasing mandibular resistance against stress while enabling a larger muscle mass for chewing and biting [114]. The detachment (and miniaturization) of the postdentary bones also enabled the detection of a wider range of airborne frequencies, particularly high-frequency sounds, by increasing the impedance-matching of the ossicular chain [105,106,115], whereas more basal NMC were likely adapted to hearing ground-borne vibrations [103,106].

## 8. Bony labyrinth

In mammals, the bony labyrinth is the endosseous space inside the petrosal (or periotic) bone that contains the membranous labyrinth, often referred to as the inner ear endocast. In basal NMC, there is no periotic as the bony labyrinth is divided between the prootic anteriorly and opisthotic posteriorly [103]. The fusion between these two bones happened in the common ancestor of Tritylodontidae, Brasilodontidae and Mammaliaformes [106,116,117]. In marsupials and placentals, the bony labyrinth is divided into two parts: (i) the coiled cochlear canal containing the organ of hearing; and (ii) the vestibule, which houses the balance organ, most noticeably the three semicircular canals [117]. In monotremes, the membranous cochlea is slightly coiled, but the corresponding osseous canal is not, resulting in a curved cochlear canal with a bulbous apex [118]. The fossil record indicates that coiling of the cochlear canal occurred convergently in the marsupial and placental lineages [117–119]. Early Triassic NMC (e.g. *Thrinaxodon*) had a very short cochlear canal, often referred to as the cochlear recess, which is difficult to distinguish from the vestibule [103,120]. The cochlear recess became increasingly elongated and distinct from the vestibule in the Probainognathia, to the point that the cochlear canal of prozostrodontian NMC is virtually indistinguishable to that of NMM [103,121]. Cochlear elongation and coiling are an adaptation to hearing a broader range of frequencies in tetrapods [118]. Changes in the geometry of the semicircular canals were also traced in NMC and mammaliaforms by Araújo *et al*. [10]. They found that a reduction in size and increase in general slenderness of the canals in the prozostrodontian NMC are correlated to changes in the viscosity of the liquid that fills up the inner ear (endolymph) because of endothermy. As metabolic rate and body temperature increased suddenly about 233 Ma, the endolymph became less viscous and the semicircular canal shapes had to adapt so the balance organ could remain functional. Accordingly, they concluded that mammalian endothermy originated around 233 Ma in the Prozostrodontia [10].

## 9. Discussion: cranial evolution and the origin of mammalian defining soft tissue features

**Figure 3.**
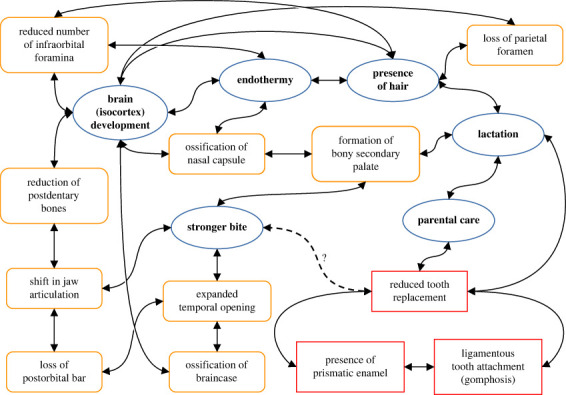
Network diagram linking the concepts of ‘mammalness’ described in the text. Soft tissue, physiological and behavioural traits in blue (ellipse) and bold text, osteological traits in orange (rounded box) and dental traits in red (square box). (Online version in colour.)

The most striking aspects of the review of the recent literature provided above are that (i) craniodental evolution is deeply interconnected with soft tissue and behavioural mammalian defining features (figure 3) and (ii) most of the changes towards ‘mammalness’ in NMC are concentrated in the Probainognathia, particularly in and around the root of the subclade Prozostrodontia (table 1).

**Table 1.** Summary of cranial characters, their soft tissue and/or behavioural correlates and the clade in which these characters probably originated (figure 1).

craniodental trait	behavioural and/or soft tissue correlate	clade of origin
secondary palate	mastication, lactation, endothermy	Epicynodontia
masseteric fossa enlarged	mastication, endothermy	Epicynodontia
differentiation of masseter and temporalis muscles	mastication, endothermy	Epicynodontia
rod-like postdentary bones	hearing?, mastication?	Eucynodontia
loss of parietal foramen	hair, lactation, endothermy, *Msx2*	Probainognathia
elongated cochlear canal	hearing, parental care	Probainognathia
dual jaw joint	mastication?	Prozostrodontia + *Probainognathus*
infraorbital foramen	hair	Prozostrodontia + *Probainognathus*
loss of postorbital bar	mastication?	Prozostrodontia
braincase simplification	isocortex?	Prozostrodontia
enlarged orbitosphenoid	olfaction, mastication?	Prozostrodontia
enlarged olfactory bulbs	olfaction, isocortex?	Prozostrodontia
smaller and slender semicircular canals	endothermy	Prozostrodontia
exposed cerebellum	hair, lactation, endothermy, *Msx2*	Prozostrodontia
circumorbital simplification	mastication?	Prozostrodontia more derived than *Prozostrodon*
dental occlusion	mastication, endothermy	Tritylodontidae + Mammaliaformes
alisphenoid (enlarged epipterygoid)	isocortex?, mastication?	Tritylodontidae + Mammaliaformes
periotic	hearing?	Tritylodontidae + Mammaliaformes
promontorium	hearing?	Mammaliaformes
bulbous braincase	isocortex?	Mammaliaformes
diphyodonty	lactation, mastication	Mammaliaformes
pterygoid muscle inserting on the dentary	mastication	Mammaliaformes
ossicular chain detachment	hearing, mastication, *Msx2*?	Mammalia and Mammaliaformes (homoplastic)
rhinal fissure	isocortex	Mammalia (homoplastic)
cochlear coiling	hearing	Mammalia (homoplastic)
ossified turbinates	endothermy, olfaction	Mammalia?
cribriform plate	olfaction	Mammalia?

An insulating coat is the most reliable evidence of endothermy [47]; however, very little direct evidence of preserved integument exists for synapsids. Known examples support the presence of reptile-like scales in the Carboniferous and Permian pelycosaurs [122–124] and that of mamillated skin (covered in round protuberances), without hair nor scales, in some Permian and Triassic non-cynodont therapsids [125,126], whereas Mesozoic mammals (and perhaps NMM) had mammal-like hairs [127–129]. However, evidence for the condition in the intermediate NMC remains elusive. The presence of an enlarged, mammal-like IOF in prozostrodontian NMC supports that they were capable of whisking [71], and thus possessed the genes to produce hair [7,66,67]. The evolution of sensory hair also probably played an important role in the origin of the isocortex [130]. Although its evolutionary origin is uncertain, it has been speculated that a six-layered isocortex first appeared as a result of improved auditory capabilities (linked to the reduction of postdentary bones) and tactile sensations (development of whiskers), which would have increased the sensory input and stimulated the development of the somatosensory regions of the cortex [53,83,96,131,132].

An origin of endothermy in prozostrodontian NMC is also supported by the study of synapsids semicircular canals adaptations to the endolymph viscosity [10]. The loss of the parietal foramen in the Probainognathia also bolsters this scenario, as the pineal eye plays a crucial role in making thermoregulatory decisions in modern ectotherms [133]. In addition, experiments on modern mice have shown that a gene which controls the presence of a parietal foramen (*Msx2*) controls the maintenance of fur coverage too. When a parietal foramen is absent (the normal condition for mammals), fur coverage is present, but when the gene is knocked-out, a parietal foramen is opened and hair follicles fail to be maintained [78,134]. These support that the loss of the parietal foramen in probainognathians is probably an osteological proxy for the appearance of fur coverage and endothermy [8]. Mice with knockout *Msx2* have a smaller vermis of the cerebellum, a condition similar to basal NMC, whereas the Probainognathia (particularly the Prozostrodontia) have a cerebellum large enough that the vermis is exposed [8,94]. This further supports that a mutation of *Msx2* was already present in the probainognathian NMC.

Fascinatingly, *Msx2* also plays a role in the development of lactation, as knockout mice are not only hairless, but also fail to display mammary glands [78]. This is consistent with the long standing and well-supported hypothesis that mammary glands are modified apocrine glands, which are tightly associated with hair follicles [4–6]. These events happen as if a significant mutation of *Msx2* occurred approximately 240–230 Ma, abruptly increasing the ‘mammalness’ of the prozostrodontian NMC.

It should be noted, however, that the origin of the bony secondary palate in Epicynodontia was a necessary prerequisite for the formation of a vacuum necessary for suckling [48], and (assuming oviparity in NMC) mammary glands might have been used originally to moisten the leathery eggshells [4]. As such, the origins of nutritive lactation, mammary glands and the secondary palate do not necessarily have to be tightly synchronous events. In fact, the late origin of diphyodonty and delayed incisor eruption in NMM [83,135], and the discovery of an adult tritylodontid preserved with approximately 38 perinates with erupted teeth [136], strongly suggests that NMC perinates were too numerous to be fed with milk, and were already capable of processing solid food [25]. This implies that NMC reproductive biology at the very root of the Mammaliaformes was still essentially reptilian, and that NMC probably did not suckle their young. Parental care (perhaps derived from opportunistic shelter-sharing [25]) seems to have been the norm for most synapsids well before the origin of lactation as abundant evidence of aggregations involving different age classes (sometimes found in burrows) have been discovered [123,136–139]. The gradual elongation of the cochlear recess in probainognathian NMC suggests that communication between parents and juvenile and social complexity increased in this lineage [140,141].

The evolution of endothermy (and thus hair) has also been tightly linked to that of miniaturization and nocturnality, as it is more difficult to maintain a constant elevated body temperature under those conditions for an ectotherm [9,24,142,143]. Miniaturization and adaptations to nocturnality both happened in probainognathian NMC [82,144] and triggered a somatosensory revolution that deeply modified their skull as it affected the anatomy of the sense organs and nervous system [53,131,144]. With increased nocturnality, sight lost its importance (loss of the sclerotic ring, photoreceptive function of the pineal organ and trichromatic vision) and was compensated by improved hearing (elongation of the cochlear recess, fusion of the periotic, origin of the promontorium), touch (origin of whiskers) and smell (enlargement of olfactory bulbs and ossification of the cribriform plate) [7,8,53,103,105,106,117,131,132,145]. Meanwhile, adaptations to high-frequency hearing accompanied miniaturization, as the incidental shrinking in size of NMC vocal organs, tympanic membrane and interaural distance impeded the production and proper hearing of low-frequency sounds [96,115,146]. This new somatosensory input fuelled the enlargement of the cerebral hemispheres, which gave its bulbous aspect to the mammalian brain case [53]. Incidentally, a larger brain generates more heat, which, in turn, helps sustaining a higher metabolic rate [24,85].

The temporalis muscle significantly grew in size in NMC, to the point that the parietal foramen became slit-like or was lost in adult individuals of some non-probainognathian NMC [8,76]. As both the braincase expanded and the jaw adductor muscles grew larger, the lateral walls of the braincase became ideal points of attachment for muscles, which selected towards a better-ossified braincase (enlarged orbitosphenoid and alisphenoid) in probainognathian NMC [89]. The same taxa also lost their postorbital bar as chewing became more intensive (and the temporalis muscle enlarged correspondingly), possibly in correlation with a change in orbit orientation [58,59]. On the lower jaw, the postdentary bones were pushed caudally and reduced in size by the growing number of muscle fibres that attached to the dentary, and the effect of miniaturization [114,144]. Noticeably, the homeogene *Msx2* is also involved in the morphogenesis of inner ear ossicles and their migration to the middle ear during ontogeny [147].

In parallel, the base of the jugal arch and masseteric fossa of the dentary became broader in NMC for attachment of the masseter muscle [13,114]. This muscle provides chewing force and encloses the mouth cavity laterally by forming the cheeks, whereas the bony secondary palate closes the mouth cavity dorsally. This compartmentation facilitates mastication by preventing the bolus from leaving the mouth cavity during chewing. Additionally, longer periods of mastication are promoted through separating the chewing and breathing functions of the snout [24]. As chewing became increasingly more important, dental morphology became more complex, and replacement became limited to enable more precise and efficient occlusion in late Triassic and early Jurassic Brasilodontidae, Tritylodontidae and Mammaliaformes [31,83]. Mammaliaforms more derived than morganucodontids subsequently lost the transverse process of the pterygoid and paracanine fossa. All these events improved food-processing capabilities, allowing for an elevated metabolic rate [24]. In this case, dentition (molars and premolars) become the main occlusal guides during mastication, which is demonstrated by the spatial consistency in the development of wear facets. Meanwhile, the cartilaginous structures of the now isolated nasal cavity began ossifying, ultimately resulting in the origin of the bony olfactory and respiratory turbinates that further improved olfaction and retention of heat and moisture in high metabolism mammals [32,33,49–51].

## 10. Conclusion

Endothermy, hair, lactation, an isocortex and parental care are considered the quintessential defining features of mammals [8,24,53,137]. The soft tissue and behavioural nature of these traits make them difficult to trace in the fossil record, but the morphological changes in the craniodental anatomy of mammals and their ancestors discussed above (e.g. transition from quadrate-articular to squamosal-dentary jaw joint, reduction in number of replacement tooth generations, ossification of the turbinates and braincase, loss of parietal foramen, etc.) offer a unique window into their evolutionary origin in the synapsid lineage. Here, we stress how strongly interconnected all these soft tissue, behavioural, osteological and dental characters are. This deep intermingling of traits has been perceived as an almost impregnable network of complexity preventing reasonable resolution or accuracy on the origin of the defining soft tissue and behavioural features of mammals from the fossil record. This resulted in a complex evolutionary scenario of highly interrelated characters evolving altogether in a slow, gradual and stepwise process [9,24,53,143]. By contrast, this review of recently studied osteological traits enables the origins of discrete major evolutionary events to be accurately pinpointed, e.g. the origin of endothermy, hair and mammary glands in probainognathian NMC, between 240 and 230 Ma [7,8,10], and the origin of lactation in NMM about 200 Ma [6,24,40]. The origin of parental care remains debated [25], but was probably present in a primitive form at the very root of the synapsid clade, 320 Ma [138]. Most of these events seem concentrated in and around the root of the clade Prozostrodontia (table 1). It is hypothesized that this clade underwent an important mutation of homeogene *Msx2*, which plays a role in the development of mammary glands, middle ear ossicles, cranial morphogenesis and hair follicle maintenance [7,8,78–81,147]. This would account for the seemingly quick evolution of ‘mammalness’ in these early Probainognathia.

## Data Availability

This article has no additional data.

## References

[1] Rowe T. 1988 Definition, diagnosis, and origin of Mammalia. J. Vertebr. Paleontol. **8**, 241-264. (10.1080/02724634.1988.10011708)

[2] Aboitiz F. 1992 The evolutionary origin of the mammalian cerebral cortex. Biol. Res. **25**, 41-49. 1341579

[3] Aboitiz F, Morales D, Montiel J. 2003 The evolutionary origin of the mammalian isocortex: towards an integrated developmental and functional approach. Behav. Brain Sci. **26**, 535-552. (10.1017/S0140525X03000128) 15179935

[4] Oftedal OT. 2002 The mammary gland and its origin during synapsid evolution. J. Mammary Gland Biol. Neoplasia **7**, 225-252. (10.1023/A:1022896515287) 12751889

[5] Oftedal OT. 2020 The evolution of lactation in mammalian species. In Milk, mucosal immunity and the microbiome: impact on the neonate (eds P Ogra, W Walker, B Lönnerdal), pp. 1-10. Basel, Switzerland: S. Karger AG. 10.1159/000505577 32155641

[6] Lefèvre CM, Sharp JA, Nicholas KR. 2010 Evolution of lactation: ancient origin and extreme adaptations of the lactation system. Annu. Rev. Genomics Hum. Genet. **11**, 219-238. (10.1146/annurev-genom-082509-141806) 20565255

[7] Benoit J, Manger PR, Rubidge BS. 2016 Palaeoneurological clues to the evolution of defining mammalian soft tissue traits. Sci. Rep. **6**, 25604. (10.1038/srep25604) 27157809 PMC4860582

[8] Benoit J, Abdala F, Manger PR, Rubidge BS. 2016 The sixth sense in mammalian forerunners: variability of the parietal foramen and the evolution of the pineal eye in South African Permo-Triassic eutheriodont therapsids. Acta Palaeontol. Polonica **61**, 777-789. (10.4202/app.00219.2015)

[9] Benton MJ. 2021 The origin of endothermy in synapsids and archosaurs and arms races in the Triassic. Gondwana Res. **100**, 261-289. (10.1016/j.gr.2020.08.003)

[10] Araújo R et al*.* 2022 Inner ear biomechanics reveals a Late Triassic origin for mammalian endothermy. Nature **613**, E2. (10.1038/s41586-022-04963-z) 35859179

[11] Ruta M, Botha-Brink J, Mitchell SA, Benton MJ. 2013 The radiation of cynodonts and the ground plan of mammalian diversity. Proc. R. Soc. B **280**, 20131865. (10.1098/rspb.2013.1865) PMC3768321 23986112

[12] Romer AS, Price LW. 1940 Review of the Pelycosauria. Spec. Pap. Geol. Soc. Amer. **28**, 1-538. (10.1130/SPE28-p1)

[13] Abdala F, Damiani R. 2004 Early development of the mammalian superficial masseter muscles in cynodonts. Palaeontol. afr. **40**, 23-29.

[14] Abdala F, Jasinoski SC, Fernandez V. 2013 Ontogeny of the Early Triassic cynodont *Thrinaxodon liorhinus* (Therapsida): dental morphology and replacement. J. Vertebr. Paleontol. **33**, 1408-1431. (10.1080/02724634.2013.775140)

[15] Norton LA, Abdala F, Rubidge BS, Botha J. 2020 Tooth replacement patterns in the Early Triassic epicynodont *Galesaurus planiceps* (Therapsida, Cynodontia). PLoS ONE **15**, e0243985. (10.1371/journal.pone.0243985) 33378326 PMC7773207

[16] Norton LA, Abdala F, Rubidge BS, Botha J. 2021 Tooth replacement in the non-mammalian cynodont *Cynosaurus suppostus* (Therapsida) from the late Permian of South Africa. J. Vertebr. Paleontol. **41**, e2001650. (10.1080/02724634.2021.2001650)

[17] Hopson JA. 1964 Tooth replacement in cynodont, dicynodont and therocephalian reptiles. Proc. Zool. Soc. Lond. **142**, 625-654. (10.1111/j.1469-7998.1964.tb04632.x)

[18] Cabreira SF, Schultz CL, da Silva L, Lora LHP, Pakulski C, do Rêgo RCB, Soares MB, Meredith SM, Richter M. 2022 Diphyodont tooth replacement of *Brasilodon*—a Late Triassic eucynodont that challenges the time of origin of mammals. J. Anat. **241**, 1424-1440. (10.1111/joa.13756) 36065514 PMC9644961

[19] Martinelli AG, Corfe IJ, Gill PG, Kallonen A, Rayfield EJ, Rodrigues PG, Schultz CL, Soares MB. 2017 *Brasilodon quadrangularis*, *Brasilitherium riogranensis* and *Minicynodon maieri* (Cynodontia): taxonomy, ontogeny and tooth replacement. Paleontologia em Destaque, Boletim de Resumos XXV Congresso Brasileiro de Paleontologia **32**, 189.

[20] Martinelli AG, Gill PG, Corfe IJ, Rodrigues PG, Fonseca PH, Schultz CL, Soares MB, Rayfield EJ. 2019 Ontogeny and tooth replacement in the Brazilian cynodonts *Brasilodon quadrangularis*, *Brasilitherium riogranensis* and *Minicynodon maieri*. Reunión de Comunicaciones de la Asociación Paleontológica Argentina, Libro de Resúmenes, 62-63.

[21] Mao FY, Zheng XT, Wang XL, Wang YQ, Meng J. 2019 Evidence of diphyodonty and heterochrony for dental development in euharamiyidian mammals from Jurassic Yanliao Biota. Vertebr. PalAsiatica **57**, 51-76. (10.19615/j.cnki.1000-3118.180803)

[22] Grine FE, Vrba ES. 1980 Prismatic enamel: a pre-adaptation for mammalian diphyodonty? S. Afr. J. Sci. **76**, 139-141.

[23] Mao F, Wang Y, Meng J. 2015 A systematic study on tooth enamel microstructures of *Lambdopsalis bulla* (Mulituberculate, Mammalia) - implications for multituberculate biology and phylogeny. PLoS ONE **10**, e0128243. (10.1371/journal.pone.0128243) 26020958 PMC4447277

[24] Kemp TS. 2005 The origin and evolution of mammals. Oxford, UK: Oxford University Press.

[25] Benoit J. 2019 Parental care or opportunism in South African Triassic cynodonts? S. Afr. J. Sci. **115**, 5589. (10.17159/sajs.2019/5589)

[26] Bertin TJC, Thivichon-Prince B, LeBlanc ARH, Caldwell MW, Viriot L. 2018 Current perspectives in tooth implantation, attachment, and replacement in Amniota. Front. Physiol. **9**, 1630. (10.3389/fphys.2018.01630) 30519190 PMC6258785

[27] LeBlanc ARH, Brink KS, Whitney MR, Abdala F, Reisz RR. 2018 Dental ontogeny in extinct synapsids reveals a complex evolutionary history of the mammalian tooth attachment system. Proc. R. Soc. B **285**, 20181792. (10.1098/rspb.2018.1792) PMC6235047 30404877

[28] Crompton AW, Parker P. 1978 Evolution of the mammalian masticatory apparatus. Am. Sci. **66**, 192-201. 646211

[29] Crompton AW. 1981 The origin of mammalian occlusion. In Orthodontics (ed. HG Barrer), pp. 3-18. Philadelphia, PA: University of Pennsylvania Press.

[30] Ungar PS. 2010 Mammal teeth: origin, evolution, and diversity. Baltimore, MD: The Johns Hopkins University Press.

[31] Grossnickle DM, Weaver LN, Jäger KRK, Schultz JA. 2022 The evolution of anteriorly directed molar occlusion in mammals. Zool. J. Linn. Soc. **194**, 349-365. (10.1093/zoolinnean/zlab039)

[32] Crompton AW, Musinsky C, Owerkowicz T. 2015 Evolution of the mammalian nose. In Great transformations in vertebrate evolution (eds KP Dial, N Shubin, EL Brainerd), pp. 189-203. Chicago, IL: University of Chicago Press.

[33] Crompton AW, Owerkowicz T, Bhullar BAS, Musinsky C. 2017 Structure of the nasal region of non-mammalian cynodonts and mammaliaforms: speculations on the evolution of mammalian endothermy. J. Vertebr. Paleontol. **37**, e1269116. (10.1080/02724634.2017.1269116)

[34] Kemp TS. 1979 The primitive cynodont *Procynosuchus*: functional anatomy of the skull and relationships. Phil. Trans. R. Soc. B **285**, 73-122. (10.1098/rstb.1979.0001)

[35] Rubidge BS, Sidor CA. 2001 Evolutionary patterns among Permo-Triassic therapsids. Annu. Rev. Ecol. Syst. **32**, 449-480. (10.1146/annurev.ecolsys.32.081501.114113)

[36] Pusch LC, Kammerer CF, Fröbisch J. 2019 Cranial anatomy of the early cynodont *Galesaurus planiceps* and the origin of the mammalian endocranial characters. J. Anat. **234**, 592-621. (10.1111/joa.12958) 30772942 PMC6481412

[37] Jasinoski SC, Abdala F, Fernandez V. 2015 Ontogeny of the Early Triassic cynodont *Thrinaxodon liorhinus* (Therapsida): cranial morphology. Anat. Rec. **298**, 1440-1464. (10.1002/ar.23116) 25620050

[38] Hopson JA, Kitching JW. 2001 A probainognathian cynodont from South Africa and the phylogeny of nonmammalian cynodonts. Bullet. Mus. Comp. Zool. **156**, 5-35.

[39] Martinelli AG, Kammerer CF, Melo TP, Paes Neto VD, Ribeiro AM, Da-Rosa ÁAS, Schultz CL, Soares MB. 2017 The African cynodont *Aleodon* (Cynodontia, Probainognathia) in the Triassic of southern Brazil and its biostratigraphic significance. PLoS ONE **12**, e0177948. (10.1371/journal.pone.0177948) 28614355 PMC5470689

[40] Kermack KA, Mussett F, Rigney HW. 1981 The skull of *Morganucodon*. Zool. J. Linn. Soc. **71**, 1-158. (10.1111/j.1096-3642.1981.tb01127.x)

[41] Rougier GW, Apesteguía SG, Gaetano LC. 2011 Highly specialized mammalian skulls from the Late Cretaceous of South America. Nature **479**, 98-102. (10.1038/nature10591) 22051679

[42] Wallace RVS, Martínez R, Rowe T. 2019 First record of a basal mammaliamorph from the early Late Triassic Ischigualasto formation of Argentina. PLoS ONE **14**, e0218791. (10.1371/journal.pone.0218791) 31390368 PMC6685608

[43] Thomason JJ, Russell AP. 1986 Mechanical factors in the evolution of the mammalian secondary palate: a theoretical analysis. J. Morphol. **189**, 199-213. (10.1002/jmor.1051890210) 3746918

[44] Crompton AW. 1972 Postcanine occlusion in cynodonts and tritylodonts. Bullet. Br. Mus. Geol. **21**, 29-71.

[45] Hopson JA, Barghusen H. 1986 An analysis of therapsid relationships. In The ecology and biology of mammal-like reptiles (eds N Hotton, PD MacLean, JJ Roth, EC Roth), pp. 83-106. Washington, DC: Smithsonian Institution Press.

[46] Abdala F, Jashashvili T, Rubidge BS, van den Heever J. 2014 New material of *Microgomphodon oligocynus* (Eutherapsida, Therocephalia) and the taxonomy of the southern African Bauriidae. In Early evolutionary history of the Synapsida (eds CF Kammerer, KD Angielczyk, J Fröbisch), pp. 209-231. Dordrecht, The Netherlands: Springer.

[47] Bennett AF, Ruben JA. 1986 The metabolic and thermoregulatory status of therapsids. In The ecology and biology of mammal-like reptiles (eds N Hotton, PD MacLean, JJ Roth, EC Roth), pp. 207-218. Washington, DC: Smithsonian Institution Press.

[48] Zhou CF, Bhullar BAS, Neander AI, Martin T, Luo ZX. 2019 New Jurassic mammaliaform sheds light on early evolution of mammal-like hyoid bones. Science **365**, 276-279. (10.1126/science.aau9345) 31320539

[49] Hillenius WJ. 1994 Turbinates in therapsids: evidence for endothermy in mammal-like reptiles. Evolution **48**, 207-229. (10.1111/j.1558-5646.1994.tb01308.x) 28568303

[50] Hillenius WJ, Ruben JA. 2004 The evolution of endothermy in terrestrial vertebrates: who? when? why? Physiol. Biochem. Zool. **77**, 1019-1042. (10.1086/425185) 15674773

[51] Owerkowicz T, Musinsky C, Middleton KM, Crompton AW. 2015 Respiratory turbinates and the evolution of endothermy in mammals and birds. In Great transformations in vertebrate evolution (eds KP Dial, N Shubin, EL Brainerd), pp. 143-166. Chicago, IL: University of Chicago Press.

[52] Ruf I, Maier W, Rodrigues PG, Schultz CL. 2014 Nasal anatomy of the non-mammaliaform cynodont *Brasilitherium riograndensis* (Eucynodontia, Therapsida) reveals new insight into mammalian evolution. Anat. Rec. **297**, 2018-2030. (10.1002/ar.23022) 25312362

[53] Rowe TB, Macrini TE, Luo ZX. 2011 Fossil evidence on the origin of the mammalian brain. Science **332**, 955-957. (10.1126/science.1203117) 21596988

[54] Rowe TB, Shepherd GM. 2016 Role of ortho-retronasal olfaction in mammalian cortical evolution. J. Comp. Neurol. **524**, 471-495. (10.1002/cne.23802) 25975561 PMC4898483

[55] Newham E, Gill PG, Corfe IJ. 2022 New tools suggest a middle Jurassic origin for mammalian endothermy. Bioessays **44**, 2100060. (10.1002/bies.202100060) 35170781

[56] Sidor CA. 2001 Simplification as a trend in synapsid cranial evolution. Evolution **55**, 1419-1442. (10.1111/j.0014-3820.2001.tb00663.x) 11525465

[57] Kerber L, Martinelli AG, Rodrigues PG, Ribeiro AM, Schultz CL, Soares MB. 2020 New record of *Prozostrodon brasiliensis* (Eucynodontia: Prozostrodontia) from its type-locality (Upper Triassic, Southern Brazil): comments on the endocranial morphology. Revista Brasileira de Paleontol. **23**, 259-269. (10.4072/rbp.2020.4.04)

[58] Heesy CP. 2005 Function of the mammalian postorbital bar. J. Morphol. **264**, 363-380. (10.1002/jmor.10334) 15844100

[59] Heesy CP, Hall MI. 2010 The nocturnal bottleneck and the evolution of mammalian vision. Brain Behav. Evol. **75**, 195-203. (10.1159/000314278) 20733295

[60] Van Valen L. 1960 Therapsids as mammals. Evolution **14**, 304-313. (10.2307/2405973)

[61] Benoit J, Kruger A, Jirah S, Fernandez V, Rubidge BS. 2021 Palaeoneurology and palaeobiology of the dinocephalian therapsid *Anteosaurus magnificus*. Acta Palaeontol. Polonica **66**, 29-39. (10.4202/app.00800.2020)

[62] Bonnan MF. 2016 The bare bones: an unconventional evolutionary history of the skeleton. Bloomington, IN: Indiana University Press.

[63] Watson DMS. 1931 On the skeleton of a bauriamorph reptile. Proc. Zool. Soc. Lond. **101**, 1163-1205. (10.1111/j.1096-3642.1931.tb01056.x)

[64] Lingham-Soliar T. 2014 The vertebrate integument, volume 1: origin and evolution. Berlin, Germany: Springer.

[65] Estes R. 1961 Cranial anatomy of the cynodont reptile *Thrinaxodon liorhinus*. Bullet. Mus. Comp. Zool. **125**, 165-180.

[66] Benoit J, Ruf I, Miyamae JA, Fernandez V, Rodrigues PG, Rubidge BS. 2020 The evolution of the maxillary canal in Probainognathia (Cynodontia, Synapsida): reassessment of the homology of the infraorbital foramen in mammalian ancestors. J. Mamm. Evol. **27**, 329-348. (10.1007/s10914-019-09467-8)

[67] Miyamae JA, Bhullar BAS. 2017 Comparative morphology of the trigeminal canal and a scenario for the evolution of facial musculature in mammals. J. Vertebr. Paleontol. Program Abstracts **2017**, 164.

[68] Franco AS, Müller RT, Martinelli AG, Hoffman CA, Kerber L. 2021 The nasal cavity of two traversodontid cynodonts (Eucynodontia, Gomphodontia) from the Upper Triassic of Brazil. J. Paleontol. **95**, 845-860. (10.1017/jpa.2021.6)

[69] Muchlinski MN. 2008 The relationship between the infraorbital foramen, infraorbital nerve, and maxillary mechanoreception: implications for interpreting the paleoecology of fossil mammals based on infraorbital foramen size. Anat. Rec. **291**, 1221-1226. (10.1002/ar.20742) 18780305

[70] Muchlinski MN. 2010 Ecological correlates of infraorbital foramen area in primates. Am. J. Biol. Anthropol. **141**, 131-141. (10.1002/ajpa.21137) 19701916

[71] Muchlinski MN, Wible JR, Corfe I, Sullivan M, Grant RA. 2020 Good vibrations: the evolution of whisking in small mammals. Anat. Rec. **303**, 89-99. (10.1002/ar.23989) 30332721

[72] Edinger T. 1933 Die Foramina parietalia der Säugetiere. Zeitschrift für Anatomie und Entwicklungsgeschichte **102**, 266-289. (10.1007/BF02134538)

[73] Edinger T. 1955 The size of parietal foramen and organ in reptiles. Bullet. Mus. Comp. Zool. **114**, 1-34.

[74] Eakin RM. 1973 The third eye. Berkeley, CA: University of California Press.

[75] Quay WB. 1979 The parietal eye-pineal complex. In *Biology of the Reptilia*, *volume 9: neurology A* (eds C Gans, RG Northcutt, P Ulinski), pp. 245-406. London, UK: Academic Press.

[76] Roth JJ, Roth EC, Hotton N. 1986 The parietal foramen and eye: their function and fate in therapsids. In The ecology and biology of mammal-like reptiles (eds N Hotton, PD MacLean, JJ Roth, EC Roth), pp. 173-184. Washington, DC: Smithsonian Institution Press.

[77] Benoit J, Abdala F, Van den Brandt MJ, Manger PR, Rubidge BS. 2015 Physiological implications for the abnormal absence of the parietal foramen in a late Permian cynodont (Therapsida). Sci. Nat. **102**, 69. (10.1007/s00114-015-1321-4) 26538062

[78] Satokata I et al. 2000 Msx2 deficiency in mice causes pleitropic defects in bone growth and ectodermal organ formation. Nat. Genet. **24**, 391-395. (10.1038/74231) 10742104

[79] Morriss-Kay G. 2001 Derivation of the mammalian skull vault. J. Anat. **199**, 143-151. (10.1017/S0021878201008093) 11523816 PMC1594961

[80] Garcia-Miñaur S, Mavrogiannis LA, Rannan-Eliya SV, Hendry MA, Liston WA, Porteous MEM, Wilkie AOM. 2003 Parietal foramina with cleidocranial dysplasia is caused by mutation in MSX2. Eur. J. Hum. Genet. **11**, 892-895. (10.1038/sj.ejhg.5201062) 14571277

[81] Roybal PG, Wu NL, Sun J, Ting MC, Schafer CA, Maxson RE. 2010 Inactivation of Msx1 and Msx2 in neural crest reveals an unexpected role in suppressing hetertopic bone formation in the head. Dev. Biol. **343**, 28-39. (10.1016/j.ydbio.2010.04.007) 20398647 PMC3279331

[82] Angielczyk KD, Schmitz L. 2014 Nocturnality in synapsids predates the origin of mammals by over 100 million years. Proc. R. Soc. B **281**, 20141642. (10.1098/rspb.2014.1642) PMC4173690 25186003

[83] Kielan-Jaworowska Z, Cifelli RL, Luo ZX. 2004 Mammals from the age of dinosaurs: origins, evolution, and structure. New York, NY: Columbia University Press.

[84] Hopson JA. 1964 The braincase of the advanced mammal-like reptile *Bienotherium*. Postilla **87**, 1-30.

[85] Benoit J, Fernandez V, Manger PR, Rubidge BS. 2017 Endocranial casts of pre-mammalian therapsids reveal an unexpected neurological diversity at the deep evolutionary root of mammals. Brain Behav. Evol. **90**, 311-333. (10.1159/000481525) 29130981

[86] Crompton AW, Musinsky C, Rougier GW, Bhullar BAS, Miyamae JA. 2018 Origin of the lateral wall of the mammalian skull: fossils, monotremes and therians revisited. J. Mamm. Evol. **25**, 301-313. (10.1007/s10914-017-9388-7)

[87] Gow CE. 1986 The side wall of the braincase in cynodont therapsids, and a note on the homology of the mammalian promontorium. South Afr. J. Zool. **21**, 136-148. (10.1080/02541858.1986.11447970)

[88] Sues HD. 1986 The skull and dentition of two tritylodontid synapsids from the Lower Jurassic of western North America. Bullet. Mus. Comp. Zool. **151**, 217-268.

[89] Benoit J, Jasinoski SC, Fernandez V, Abdala F. 2017 The mystery of a missing bone: revealing the orbitosphenoid in basal Epicynodontia (Cynodontia, Therapsida) through computed tomography. Sci. Nat. **104**, 66. (10.1007/s00114-017-1487-z) 28721557

[90] Koyabu D, Maier W, Sánchez-Villagra MR. 2012 Paleontological and developmental evidence resolve the homology and dual embryonic origin of a mammalian skull bone, the interparietal. Proc. Natl Acad. Sci. USA **109**, 14 075-14 080. (10.1073/pnas.1208693109) PMC3435230 22891324

[91] Gaetano LC et al. 2022 A new cynodont from the Upper Triassic Los Colorados Formation (Argentina, South America) reveals a novel paleobiogeographic context for mammalian ancestors. Sci. Rep. **12**, 6451. (10.1038/s41598-022-10486-4) 35468982 PMC9038739

[92] Macrini TE, Rougier GW, Rowe T. 2007 Description of a cranial endocast from the fossil mammal *Vincelestes neuquenianus* (Theriiformes) and its relevance to the evolution of endocranial characters in therians. Anat. Rec. **290**, 875-892. (10.1002/ar.20551) 17506058

[93] Hoffman S, O'Connor PM, Kirk EC, Wible JR, Krause DW. 2014 Endocranial and inner ear morphology of *Vintana sertichi* (Mammalia, Gondwanatheria) from the Late Cretaceous of Madagascar. J. Vertebr. Paleontol. **34**, 110-137. (10.1080/02724634.2014.956878)

[94] Rodrigues PG, Martinelli AG, Schultz CL, Corfe IJ, Gill PG, Soares MB, Rayfield EJ. 2019 Digital cranial endocast of *Riograndia guaibensis* (Late Triassic, Brazil) sheds light on the evolution of the brain in non-mammalian cynodonts. Hist. Biol. **31**, 1195-1212. (10.1080/08912963.2018.1427742)

[95] Rakic P. 2009 Evolution of the neocortex: perspective from developmental biology. Nat. Rev. Neurosci. **10**, 724-735. (10.1038/nrn2719) 19763105 PMC2913577

[96] Rowe TB. 2017 The emergence of mammals. In Evolution of nervous systems (ed. JH Kaas), pp. 1-52. New York, NY: Academic Press.

[97] Huttenlocker AK, Grossnickle DM, Kirkland JI, Schultz JA, Luo ZX. 2018 Late-surviving stem mammal links the lowermost Cretaceous of North America and Gondwana. Nature **558**, 108-112. (10.1038/s41586-018-0126-y) 29795343

[98] Crompton AW, Hylander WL. 1986 Changes in mandibular function following the acquisition of a dentary-squamosal jaw articulation. In The ecology and biology of mammal-like reptiles (eds N Hotton, PD MacLean, JJ Roth, EC Roth), pp. 263-282. Washington, DC: Smithsonian Institution Press.

[99] Luo ZX. 2007 Transformation and diversification in early mammal evolution. Nature **450**, 1011-1019. (10.1038/nature06277) 18075580

[100] Allin EF. 1975 Evolution of the mammalian middle ear. J. Morphol. **147**, 403-437. (10.1002/jmor.1051470404) 1202224

[101] Allin EF, Hopson JA. 1992 Evolution of the auditory system in Synapsida (‘mammal-like reptiles' and primitive mammals) as seen in the fossil record. In The evolutionary biology of hearing (eds DB Webster, AN Popper, RR Fay), pp. 587-614. Berlin, Germany: Springer.

[102] Kemp TS. 2016 Non-mammalian synapsids: the beginning of the mammal line. In Evolution of the vertebrate ear (eds JA Clack, RR Fay, AN Popper), pp. 107-137. Cham, Switzerland: Springer.

[103] Luo ZX. 2011 Developmental patterns in Mesozoic evolution of mammal ears. Ann. Rev. Ecol. Evol. Syst. **42**, 355-380. (10.1146/annurev-ecolsys-032511-142302)

[104] Gaetano LC, Abdala F. 2015 The stapes of gomphodont cynodonts: insights into the middle ear structure of non-mammaliaform cynodonts. PLoS ONE **10**, e0131174. (10.1371/journal.pone.0131174) 26176619 PMC4503721

[105] Ekdale EG. 2016 The ear of mammals: from monotremes to humans. In Evolution of the vertebrate ear (eds JA Clack, RR Fay, AN Popper), pp. 175-206. Cham, Switzerland: Springer.

[106] Luo ZX, Schultz JA, Ekdale EG. 2016 Evolution of the middle and inner ears of Mammaliaforms: the approach to mammals. In Evolution of the vertebrate ear (eds JA Clack, RR Fay, AN Popper), pp. 139-174. Cham, Switzerland: Springer.

[107] Maier W, Ruf I. 2016 Evolution of the mammalian middle ear: a historical review. J. Anat. **228**, 270-283. (10.1111/joa.12379) 26397963 PMC4718169

[108] Hopson JA. 1987 The mammal-like reptiles. A study of transitional fossils. Am. Biol. Teacher **49**, 16-26. (10.2307/4448410)

[109] Luo ZX, Chen P, Li G, Chen M. 2007 A new eutriconodont mammal and evolutionary development in early mammals. Nature **446**, 288-293. (10.1038/nature05627) 17361176

[110] Mao F, Hu Y, Li C, Wang Y, Chase MH, Smith AK, Meng J. 2020 Integrated hearing and chewing modules decoupled in Cretaceous stem therian mammal. Science **367**, 305-308. (10.1126/science.aay9220) 31806694

[111] Wang J, Wible JR, Guo B, Shelley SL, Hu H, Bi S. 2021 A monotreme-like auditory apparatus in a Middle Jurassic haramiyidan. Nature **590**, 279-283. (10.1038/s41586-020-03137-z) 33505017

[112] Le Maître A, Grunstra NDS, Pfaff C, Mitteroecker P. 2020 Evolution of the mammalian ear: an evolvability hypothesis. Evol. Biol. **47**, 187-192. (10.1007/s11692-020-09502-0) 32801400 PMC7399675

[113] Schultz JA. 2020 Eat and listen-how chewing and hearing evolved. Science **367**, 244-247. (10.1126/science.aba3808) 31949065

[114] Lautenschlager S, Gill P, Luo ZX, Fagan MJ, Rayfield EJ. 2017 Morphological evolution of the mammalian jaw adductor complex. Biol. Rev. **92**, 1910-1940. (10.1111/brv.12314) 27878942 PMC6849872

[115] Manley GA. 2018 The foundations of high-frequency hearing in early mammals. J. Mamm. Evol. **25**, 155-163. (10.1007/s10914-016-9379-0)

[116] Rodrigues PG, Ruf I, Schultz CL. 2013 Digital reconstruction of the otic region and inner ear of the non-mammalian cynodont *Brasilitherium riograndensis* (Late Triassic, Brazil) and its relevance to the evolution of the mammalian ear. J. Mamm. Evol. **20**, 291-307. (10.1007/s10914-012-9221-2)

[117] Ekdale EG. 2016 Form and function of the mammalian ear. J. Anat. **228**, 324-337. (10.1111/joa.12308) 25911945 PMC4718163

[118] Ekdale EG. 2013 Comparative anatomy of the bony labyrinth (inner ear) of placental mammals. PLoS ONE **8**, e66624. (10.1371/journal.pone.0066624) 23805251 PMC3689836

[119] Luo ZX, Manley GA. 2020 Origins and early evolution of mammalian ears and hearing function. In The senses: a comprehensive reference (ed. B Grothe), pp. 207-252. Amsterdam, The Netherlands: Elsevier.

[120] Benoit J, Manger PR, Fernandez V, Rubidge BS. 2017 The bony labyrinth of the late Permian Biarmosuchia: palaeobiology and diversity in non-mammalian Therapsida. Palaeontol. afr. **52**, 58-77.

[121] Benoit J, Nxumalo M, Norton LA, Fernandez V, Gaetano LC, Rubidge B, Abdala F. 2022 Synchrotron scanning sheds new light on *Lumkuia fuzzi* (Therapsida, Cynodontia) from the Middle Triassic of South Africa and its phylogenetic placement. J. Afr. Earth Sci. **196**, 104689. (10.1016/j.jafrearsci.2022.104689)

[122] Reisz R. 1975 Pennsylvanian pelycosaurs from Linton, Ohio and Nýřany, Czechoslovakia. J. Paleontol. **49**, 522-527.

[123] Botha-Brink J, Modesto SP. 2007 A mixed-age classed ‘pelycosaur’ aggregation from South Africa: earliest evidence of parental care in amniotes? Proc. R. Soc. B **274**, 2829-2834. (10.1098/rspb.2007.0803) PMC2288685 17848370

[124] Spindler F, Werneburg R, Schneider LL, Annacker V, Rößler R. 2018 First arboreal ‘pelycosaurs’ (Synapsida: Varanopidae) from the early Permian Chemnitz Fossil Lagerstätte, SE Germany, with a review of varanopid phylogeny. PalZ **92**, 315-364. (10.1007/s12542-018-0405-9)

[125] Chudinov PK. 1968 Structure of the integuments of theromorphs. Dokl. Akad. Nauk SSSR **179**, 226-229.

[126] Smith RMH, Botha J, Viglietti PA. 2022 Taphonomy of drought afflicted tetrapods in the Early Triassic Karoo Basin, South Africa. Palaeogeogr. Palaeoclimatol. Palaeoecol. **604**, 111207. (10.1016/j.palaeo.2022.111207)

[127] Ji Q, Luo ZX, Yuan CX, Tabrum AR. 2006 A swimming mammaliaform from the Middle Jurassic and ecomorphological diversification of early mammals. Science **311**, 1123-1127. (10.1126/science.1123026) 16497926

[128] Martin T, Marugán-Lobón J, Vullo R, Martin-Abad H, Luo ZX, Buscallioni AD. 2015 A Cretaceous eutriconodont and integument evolution in early mammals. Nature **526**, 380-384. (10.1038/nature14905) 26469049

[129] Meng QJ, Grossnickle DM, Liu D, Zhang YG, Neander AI, Ji Q, Luo ZX. 2017 New gliding mammaliaforms from the Jurassic. Nature **548**, 291-296. (10.1038/nature23476) 28792929

[130] Luzzati F. 2015 A hypothesis for the evolution of the upper layers of the neocortex through co-option of the olfactory cortex developmental program. Front. Neurosci. **9**, 162. (10.3389/fnins.2015.00162) 26029038 PMC4429232

[131] Jerison HJ. 1973 Evolution of the brain and intelligence. New York, NY: Academic Press.

[132] Rowe T. 1996 Coevolution of the mammalian middle ear and neocortex. Science **273**, 651-654. (10.1126/science.273.5275.651) 8662557

[133] Ralph CL, Firth BT, Gern WA, Owens DW. 1979 The pineal complex and thermoregulation. Biol. Rev. **54**, 41-72. (10.1111/j.1469-185X.1979.tb00867.x) 375995

[134] Ferguson MWJ. 2000 A hole in the head. Nat. Genet. **24**, 330-331. (10.1038/74132) 10742087

[135] Luo ZX, Kielan-Jaworowska Z, Cifelli RL. 2004 Evolution of the dental replacement in mammals. Bullet. Carnegie Mus. Natural Hist. **36**, 159-175. (10.2992/0145-9058(2004)36[159:EODRIM]2.0.CO;2)

[136] Hoffman EA, Rowe TB. 2018 Jurassic stem-mammal perinates and the origin of mammalian reproduction and growth. Nature **561**, 104-108. (10.1038/s41586-018-0441-3) 30158701

[137] Jasinoski SC, Abdala F. 2017 Aggregations and parental care in the Early Triassic basal cynodonts *Galesaurus planiceps* and *Thrinaxodon liorhinus*. PeerJ **5**, e2875. (10.7717/peerj.2875) 28097072 PMC5228509

[138] Maddin HC, Mann A, Hebert B. 2020 Varanopid from the Carboniferous of Nova Scotia reveals evidence of parental care in amniotes. Nat. Ecol. Evol. **4**, 50-56. (10.1038/s41559-019-1030-z) 31900446

[139] Smith RMH, Angielczyk KD, Benoit J, Fernandez V. 2021 Neonate aggregation in the Permian dicynodont *Diictodon* (Therapsida, Anomodontia): evidence for a reproductive function for burrows. Palaeogeogr. Palaeoclimatol. Palaeoecol. **569**, 110311. (10.1016/j.palaeo.2021.110311)

[140] Walsh S, Barrett PM, Milner AC, Manley G, Witmer LM. 2009 Inner ear anatomy is a proxy for deducing auditory capability and behaviour in reptiles and birds. Proc. R. Soc. B **276**, 1355-1360. (10.1098/rspb.2008.1390) PMC2660952 19141427

[141] Hanson M, Hoffman EA, Norell MA, Bhullar BAS. 2021 The early origin of a birdlike inner ear and the evolution of dinosaurian movement and vocalization. Science **372**, 601-609. (10.1126/science.abb4305) 33958471

[142] McNab BK. 1983 Energetics, body size, and the limits to endothermy. J. Zool. **199**, 1-29. (10.1111/j.1469-7998.1983.tb06114.x)

[143] Lovegrove BG. 2017 A phenology of the evolution of endothermy in birds and mammals. Biol. Rev. **92**, 1213-1240. (10.1111/brv.12280) 27154039

[144] Lautenschlager S, Gill P, Luo ZX, Fagan MJ, Rayfield EJ. 2018 The role of miniaturization in the evolution of the mammalian jaw and middle ear. Nature **561**, 533-537. (10.1038/s41586-018-0521-4) 30224748

[145] Gerkema MP, Davies WIL, Foster RG, Menaker M, Hut RA. 2013 The nocturnal bottleneck and the evolution of activity patterns in mammals. Proc. R. Soc. B **280**, 20130508. (10.1098/rspb.2013.0508) PMC3712437 23825205

[146] Manley GA. 2017 Comparative auditory neuroscience: understanding the evolution and function of ears. J. Assoc. Res. Otolaryngol. **18**, 1-24. (10.1007/s10162-016-0579-3) 27539715 PMC5243258

[147] Zhang Z, Zhang X, Avniel WA, Song Y, Jones SM, Jones TA, Fermin C, Chen Y. 2003 Malleal processus brevis is dispensable for normal hearing in mice. Dev. Dyn. **227**, 69-77. (10.1002/dvdy.10288) 12701100

